# Nanomaterials and Their Recent Applications in Impedimetric Biosensing

**DOI:** 10.3390/bios13100899

**Published:** 2023-09-22

**Authors:** Zala Štukovnik, Regina Fuchs-Godec, Urban Bren

**Affiliations:** 1 Faculty of Chemistry and Chemical Engineering, University of Maribor, Smetanova Ulica 17, 2000 Maribor, Slovenia; zala.stukovnik1@um.si (Z.Š.); regina.fuchs@um.si (R.F.-G.); 2 Faculty of Mathematics, Natural Sciences and Information Technologies, University of Primorska, Glagoljaška Ulica 8, 6000 Koper, Slovenia; 3 Institute of Environmental Protection and Sensors, Beloruska ulica 7, 2000 Maribor, Slovenia

**Keywords:** impedimetric biosensor, electrochemical impedance spectroscopy, nanomaterials, metal nanoparticles, carbon nanofibers, carbon nanotubes, graphene oxide, quantum dots

## Abstract

Impedimetric biosensors measure changes in the electrical impedance due to a biochemical process, typically the binding of a biomolecule to a bioreceptor on the sensor surface. Nanomaterials can be employed to modify the biosensor’s surface to increase the surface area available for biorecognition events, thereby improving the sensitivity and detection limits of the biosensor. Various nanomaterials, such as carbon nanotubes, carbon nanofibers, quantum dots, metal nanoparticles, and graphene oxide nanoparticles, have been investigated for impedimetric biosensors. These nanomaterials have yielded promising results in improving sensitivity, selectivity, and overall biosensor performance. Hence, they offer a wide range of possibilities for developing advanced biosensing platforms that can be employed in various fields, including healthcare, environmental monitoring, and food safety. This review focuses on the recent developments in nanoparticle-functionalized electrochemical-impedimetric biosensors.

## 1. Introduction

Due to an increased demand for specialized sensors that can deliver fast and accurate readings in various research fields, the biosensor market has recently gained significant popularity. Biochemical profiling of healthy and pathological cells, clinical diagnosis, drug discovery, and simplified analyses in processes of environmental monitoring and food and beverage quality control represent just several of the applications, whereby the development of electrochemical biosensors is of interest [1,2,3,4]. Biosensors represent small and cost-effective analytical tools that convert a biological response into an electrical signal and indicate the concentration of the target analyte [5,6]. The type of analyte composition, the biologically active component, the biosensor’s design, and the transducer’s physical characteristics affect this information’s precision [6,7].

The type of transducer is crucial to developing an effective and reliable electrochemical sensor. Nanomaterials, including metal oxide nanoparticles, metal nanoparticles, carbon nanotubes, and graphene, provide some benefits in this respect and are far more desirable than conventional materials for electrochemical biosensors due to their unique electrical, chemical, and mechanical properties [8].

Moreover, the selectivity of the biological detector element opens up the prospect of developing specialized instruments for real-time investigation of complex mixtures. The major biological components include enzyme/substrate, antibody/antigen, and nucleic acid/complementary sequence pairings [9].

The advantages of biosensors include their high specificity and sensitivity, capability of rapid detection, and low cost [10]. Thin-layer chromatography (TLC), high-performance liquid chromatography combined with mass spectrometry (HPLC-MS), and capillary electrophoresis (EC) represent the three main traditional analytical techniques that also meet these requirements [11,12]. However, they also possess substantial drawbacks, including requiring highly skilled workers, expensive equipment, and large sample quantities [12]. Biosensors have become outstanding analytical tools for overcoming these limitations. In this sense, electrochemical approaches have recently received much interest in developing biosensors due to their capacity for a mass production at low cost [13,14].

Biosensors using electrochemical impedimetric spectroscopy detect the electrical impedance developed at the electrode/electrolyte interface in response to excitation by a small sinusoidal signal [6,15]. Electrochemical impedance spectroscopy (EIS) represents a promising electrochemical technique used in biosensors to characterize both biocatalytic and electrode transformations as well as the transduction of biosensing events at electrodes [16,17,18,19,20]. The EIS integrates data on materials’ capacitive and resistive properties [21,22,23]. Moreover, since it is a non-destructive technique that provides high-quality data, it forms an important tool for biosensor development [24]. The setup of an EIS system is very light weight and portable. Therefore, the analysis can also be performed outside the laboratory [24,25].

**Figure 1:**
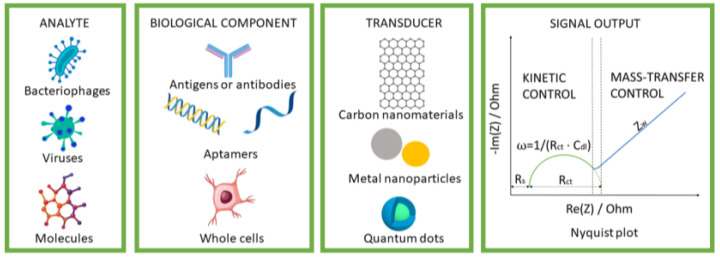
Nanomaterials and their role in the development of impedimetric biosensors.

Characterization of the modified electrodes remains the main application of EIS in the context of developing biosensors. EIS experiments can be performed during the modifications in biosensing platforms and can provide additional information about occurring events on the electrode [26]. EIS can be employed to characterize the properties of an electrode/electrolyte interface, such as charge transfer resistance (*R*_ct_), double-layer capacitance (*C*_dl_), and diffusion processes (*Z*_w_). Experimental values of the EIS measurements are usually plotted using the Nyquist plot, where the Im(Z) is plotted against the Re(Z) examined frequency range. Figure 1 demonstrates that Nyquist plots often include a semicircular and a linear portion related to the electrode’s modifying layers. The linear portion describes the diffusion-limited process, whereas the semicircular portion, visible at higher frequencies, describes the electron transfer. The *R*_ct_ at the electrode surface is calculated using the semicircular diameter, which typically increases when the analyte binds to the biorecognition component [27]. The *R*_ct_ is related to the ease with which charge is transferred across the electrode/electrolyte interface during an electrochemical reaction [28]. Investigating the impedance spectra can determine if the modification affected the charge transfer kinetics and resistance associated with the electrochemical reaction. A decrease in charge transfer resistance indicates enhanced electron transfer, while an increase indicates impeded charge transfer. Moreover, modification of the electrode surface can alter the surface area or properties that affect the formation and behavior of the electrical double layer at the electrode/electrolyte interface [29]. Investigating the impedance spectra can provide information about the changes in the *C*_dl_, indicating modifications in the surface area or in the interaction between the electrode and the electrolyte. Furthermore, the EIS can provide insight into changes in mass transport processes associated with electrode modification. If an electrode is modified to increase its surface roughness, the impedance spectra likely reflect changes in diffusion processes or reactant transport [30].

Reference standards for nanomaterial-modified electrodes are essential to ensure consistent and reliable experimental results when working with electrochemical biosensors and analyses using nanomaterial-modified electrodes [31]. Ferricyanides are a redox active species commonly applied to calibrate and test electrochemical test assemblies, often used to characterize the electrochemical properties and electron transfer kinetics of CNT-, CNF-, and graphene-modified electrodes. Their well-defined redox potentials make them suitable for testing electrode performance. The most commonly employed ferricyanide due to its stable and reversible redox reactions is [Fe(CN)_6_]^3−/4−^ [32]. Hydrogen peroxide (H_2_O_2_) can be utilized in graphene- and metal NPs-modified electrodes that can catalyze oxygen reduction or the oxidation of hydrogen peroxide. The specific metal nanoparticles such as Au, Pt, and Ag may participate in their own redox reactions. For example, silver nanoparticles can undergo redox processes involving the [Ag^+^/Ag] couple [33]. Ferrocene/ferrocenium can also be employed for graphene oxide-, CNT-, and CNF-modified electrodes [34,35]. When it comes to an understanding of the properties of specific biorecognition events, including the process of antigen or antibody capture at the electrode surface, the molecular biorecognition of certain proteins, as well as the recognition of receptors, nucleic acids, and even whole cells, EIS represents an important technique [14,36]. However, electrochemical impedimetric biosensors have the disadvantage that their performance degrades over time, and the current flow can be hindered. Several strategies have been explored to circumvent this effect and enhance the signal, such as binding to various labeling molecules, including DNA, enzymes, nanoparticles, and carbon compounds [37].

Various studies have been conducted on using nanomaterials in the production of biosensors since there are certain challenges when measuring the biomolecule’s electrochemical reactions using bare electrodes [38,39,40]. For example, many biomolecules with similar oxidation potentials may be present in biological samples, obstructing the target biomolecules’ signals. It is also possible that these biomolecule concentrations are very low to measure [41]. The selectivity of the electrode can be substantially increased if the changed surface can only bind to the target, making it more straightforward to detect the actual sample [42,43]. One way the nanoparticles can improve impedimetric biosensors is by functionalizing the surface with specific biomolecules that can selectively bind to the target analyte in a sample (Figure 1). This can increase the specificity and sensitivity of the biosensor, as the nanoparticles can act as amplifiers of the signal generated by the binding event.

Another way that nanoparticles can be used is by modifying the surface of the biosensor with a layer of nanoparticles that can enhance the binding of the biomolecules to the receptor. This can increase the surface area available for binding, leading to a stronger signal and a better detection of the analyte. Nanoparticles can also be applied as nanoelectrodes, which can increase the sensitivity of the biosensor by reducing the distance between the electrode and the analyte. This can allow for a more efficient charge transfer and faster response times [44,45]. Numerous nanomaterials are suitable for biosensing due to their distinct characteristics [46]. Nanometer-sized particles exert a large specific surface area, good biocompatibility, chemical stability, mechanical strength, and the possibility of easy functionalization [47,48]. Additionally, certain nanoparticles have the capacity to function as small conduction centers and promote electron transport [49,50,51].

To be effective in biosensing, these nanoparticles must be chemically resistant to extreme conditions, cause little disruption in the system being studied, and exhibit low or no toxicity [52]. The stability of nanoparticle biosensors must be sufficient to endure long-term storage and changing environments [53]. The important roles of nanoparticles include immobilization of biomolecules, electrochemical reactions catalyzation, enhancement of electron transfer between electrode surfaces and biomolecules, labeling of biomolecules, and even acting as reactants [54].

**Figure 2:**
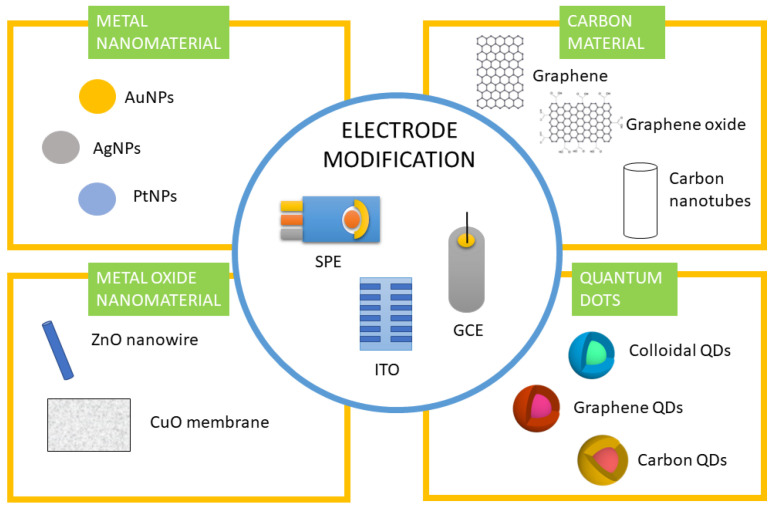
Electrodes such as screen printed electrodes (SPE), glassy carbon electrodes (GCE), and indium tin oxide (ITO) electrodes modified with different nanomaterials such as metal nanomaterials, including gold nanoparticles (AuNPs), silver nanomaterials (AgNPs), platinum nanoparticles (PtNPs), carbon materials, including graphene, graphene oxide, and carbon nanotubes, metal oxide nanomaterials, such as ZnO nanowires and CuO membranes, and quantum dots, including colloidal, graphene, and carbon quantum dots (QDs).

Lately, to enhance the performance of electrochemical biosensors, it has been proposed to modify the working electrode (WE) with nanomaterials such as nanoparticles (NPs), carbon nanotubes (CNTs), carbon nanofibers (CNFs), graphene oxide (GO), reduced graphene oxide (rGO), or quantum dots (QDs) (Figure 2) [55,56,57,58].

Metal nanoparticles, such as silver as well as gold nanoparticles, are commonly used in impedimetric biosensors due to their unique optical and electrical properties [59]. These nanoparticles exhibit a large surface-to-volume ratio, which increases their sensitivity and specificity. In addition, gold nanoparticles are biocompatible and can be easily functionalized with biological molecules, making them suitable for bioanalytical applications. Carbon-based nanoparticles, such as graphene and carbon nanotubes, are also widely applied in impedimetric biosensors. These nanoparticles possess high-electrical conductivity, large surface area, and high-mechanical strength, making them great for using electrodes in biosensors [60]. Moreover, carbon-based nanoparticles can be functionalized with biomolecules to enhance their specificity and sensitivity [61]. QDs represent semiconductor nanoparticles that exhibit unique optical and electronic properties. These nanoparticles possess high photoluminescence, tunable fluorescence, and excellent stability, making them ideal for use in biosensors. In addition, QDs have large surface areas that can be functionalized with biological molecules, which increase their specificity and sensitivity [62].

When applied in biosensors, each nanoparticle (NP) type has unique advantages and disadvantages. The selection of nanoparticles depends on the specific application and the target analyte. The choice of material depends on the specific application and the desired properties of the biosensor [63].

As is widely acknowledged, over recent years, electrodes modified with nanoparticles have become increasingly important in biosensor research [19,64,65]. The abovementioned properties make nanomaterials advantageous for developing sensitive and specific electrochemical sensors for trace-level quantification [35,66].

This review covers various nanomaterials, ranging from metal nanoparticles to 2D transition metal dichalcogenides, carbon-based materials (carbon nanotubes, carbon nanofibers, graphene oxide, graphene), and quantum dots. This review draws on the latest advances in the field by including emerging nanomaterials such as 2D transition metal dichalcogenides and quantum dots. In addition, the focus is on impedimetric biosensors using EIS as the main detection technique. Since EIS is a non-destructive method, it is particularly important for biosensor applications where sample preservation is crucial. Given the wide range of linearity and sensitivity offered by the described nanomaterial-modified impedimetric biosensors, our work will certainly find application in several areas, including medical diagnostics, environmental monitoring, and food safety.

## 2. Nanomaterials

### 2.1. Metal and Metal Oxide Nanoparticles and Two-Dimensional Transition Metal Dichalcogenides

There are several good reasons for using nanoparticles in the biosensor design, including a large surface area, a flexible surface for molecular functionalization, electrocatalytic properties, and the facilitation of a direct electron transfer [67]. Due to their resistance to oxidation and high biocompatibility, precious metals like Au, Ag, Pt, and Pd are commonly selected for electrode modification [68,69]. In addition, metal oxide nanoparticles such as NiO, ZnO, CuO, and TiO_2_ provide similar advantages. Nevertheless, they are less expensive and have simple production protocols. They have been widely used in faradaic biosensors to enable rapid electron transfer kinetics between the electrode and the active sites of biomolecules [70]. These nanoparticles can alter the sensing substrate by depositing them on the electrode surface or by combining them with the remaining elements of the constructed electrode matrix.

The large and flexible surface area of metal nanoparticles allows for a greater deposition of biomolecules during immobilization, resulting in a greater impedance response. This is one of the main purposes of using these nanoparticles [69,70].

It is also possible to improve detection performance by using two-dimensional transition metal dichalcogenides (2D TMDs), which exhibit promising features like biocompatibility, a large surface area for detection, and numerous electrochemical property-modifying possibilities [71]. Two-dimensional TMDs represent MX2-type compounds with layered structures formed by covalent bonds and weak interlayer interactions. Each MX2 monolayer consists of two layers of chalcogen atoms and a layer of a transition metal atom (M) [72]. Two-dimensional TMDs include molybdenum disulfide (MoS_2_), molybdenum diselenide (MoSe_2_), tungsten disulfide (WS_2_), tungsten diselenide (WSe_2_), hexagonal boron nitride (h-BN), borophene (2D boron), silicene (2D silicon), germanene (2D germanium), and MXenes (2D carbides/nitrides) [73]. Due to their larger surface area, 2D TMDs can immobilize large amounts of biomolecules per unit area [74].

### 2.2. Graphene and Graphene Oxide (GO)

Compared to other sensors made of nanomaterials, graphene-based biosensors exhibit several unique advantages. For example, the unique 2D structure and thickness of graphene sheets of only one atom allow each carbon atom to interact directly with the analyte, making graphene-based biosensors very sensitive to changes in environmental conditions [42]. GO represents a multilayer, oxygenated graphene sheet with a carbon-to-oxygen ratio of roughly 3:1. It contains oxygen functional groups, including epoxides, carboxyls, hydroxyls, and alcohols at the sheet’s edge and surface [42,75]. By increasing the rate of heterogeneous electron and charge transfer, oxygen functional groups on the graphene surface may make GO more water-soluble and biocompatible [42,76]. Following reduction, GO changes into rGO, which contains some residual oxygen and structural defects. Reduction yields a material with high-thermal conductivity comparable to doped conductive polymers, which is about 36 times greater than silicon and around 100 times better than GaAs [42,77]. Compared to CNTs, graphene has two major advantages in terms of its application in electrochemical sensors. Firstly, graphene is produced from graphite, a widely available and inexpensive material, and does not include any metallic impurities that would interfere with the electrochemical properties of the material. Secondly, π–π stacking and hydrophobic interactions make it simple to immobilize biomolecules on graphene. The benefits of graphene as a channel material include a decreased noise ratio, ease of functionalization, processability in solutions, and biocompatibility [42,78].

### 2.3. Carbon Nanotubes (CNTs)

Carbon nanotubes (CNTs) represent one of the most popular nanomaterials made of cylindrical structures comprising a corrugated graphene layer [79]. Due to their unique structure and nanoscale dimensions, CNTs have many properties that can be employed for chemical and biological sensing applications. As an example of a chemical sensor application, Giordano et al. [80] developed a sensor with a film of multi-walled carbon nanotubes (MWCNTs) that can be used to monitor alcohol concentration in liquid solutions such as alcoholic beverages. CNTs are structurally comparable to a sheet of graphene that has been rolled up. Single-walled carbon nanotubes (SWCNTs) and multi-walled carbon nanotubes (MWCNTs) represent the two main forms of CNTs [18]. From the perspective of biosensor analysis, both types of CNTs have their advantages. While MWCNTs exhibit excellent corrosion resistance, SWCNTs have strong elastic modulus and tensile strength [81]. Certain limitations make SWCNTs harder to function as biosensors than other materials. For example, they are very small to bind to some biomolecules, such as cells. Nevertheless, some neurotransmitters or DNA biosensors nowadays use SWCNT as a modification material [43].

CNTs exhibit good mechanical properties and have a modulus of elasticity equivalent to that of a diamond, as well as good conductivity because their sheet structure is similar to that of graphene. Moreover, this material has great potential in terms of thermal conduction. Furthermore, CNTs exhibit many advantages, such as their light weight, large specific surface area, chemical stability, and good electrochemical properties, which offer a promising research potential for biomolecular detection in medicine. The CNTs’ large specific surface area offers a variety of reactive sites that make it easier for them to interact with different biomolecules. Additionally, because CNTs’ electrical conductivity is sensitive to analyte absorption, they can also be used to create sensitive, label-free biosensors [82].

### 2.4. Carbon Nanofibers (CNFs)

Among the numerous nanomaterials, carbon nanofibers (CNFs) have become one of the most investigated areas in nanomaterial science due to their excellent biological and physicochemical properties, such as biocompatibility, large specific surface area, and easy functionalization [83]. CNFs are represented by cylindrical nanocarbon structures with different arrangements in stacked graphene sheets [84]. The cylindrical structure of CNFs can be solid or hollow, with a length of up to 10 μm and with a diameter of 10 to 500 nm [48].

Graphite, glassy carbon, carbon fibers, nanotubes, amorphous powders, and diamonds represent only a few examples of the different microstructures found in carbon materials [48,84]. CNFs are similar to CNTs in terms of electrical and mechanical properties. However, the size and layout of CNFs may be precisely adjusted. Nevertheless, the size and graphite arrangement of CNFs can be well controlled [48,85].

Furthermore, compared to other structures like CNTs, CNFs with more edges on their outer wall exhibit a better potential for electron transmission [86]. CNFs have been widely used in developing biosensors due to their unique physical and chemical properties, including excellent electrical conductivity, large surface area, biocompatibility, and the ease of production [87,88].

### 2.5. Quantum Dots (QDs)

Due to their extremely large surface area and dangling bonds, QDs represent zero-dimensional semiconductor nanoparticles with diameters typically ranging from 1 to 20 nm and many active sites [89]. Typically, QDs are made of II, VI, B, or III, V, or their alloyed semiconductor materials [90].

The popularity of QDs, including carbon quantum dots (CQDs) and graphene quantum dots (GQDs), has increased as a result of their distinctive qualities, including good biocompatibility, electrocatalytic activity, tunable size, good signal amplification, and multiplex detection capacity [91]. In contrast to the majority of carbon allotropes, such as CNTs or graphene, CDs also exhibit excellent stability, high solubility, low toxicity, amplified adsorption ability, and high-quantum yield [92]. CQDs were unintentionally discovered in 2004 while single-walled carbon nanotubes (SWCNTs) were being purified [93].

## 3. Application of Nanomaterials

### 3.1. Metal and Metal Oxide Nanoparticles and Two-Dimensional Transition Metal Dichalcogenides

Several studies have been performed using nanoscale impedimetric biosensors for detecting various analytes, such as proteins, viruses, toxins, or biomarkers (Table 1).

Nano-ZnO/CuO and nano-ZnO nitrocellulose membrane biosensors were compared by Cao et al. [94], and both were developed utilizing a straightforward and affordable sonication technique. Nano-ZnO/CuO membranes were prepared by sonicating colloidal suspensions of 1% (*w*/*v*) ZnO and 1% (*w*/*v*) CuO nanocrystals at a volume ratio of 1:2. According to impedance spectroscopy measurements, the sonication increased the output signal by a factor of more than two. To evaluate dose-dependent responses to the C-reactive protein (CRP), changes in impedance phase values at a frequency of 100 Hz were examined. The nitrocellulose membrane biosensor with 1% (*w*/*v*) nano-ZnO had an LOD of 27 pg/mL, and a biosensor with 1% (*w*/*v*) nano-ZnO/CuO nitrocellulose had an LOD of 16 pg/mL, respectively.

Roushani et al. [95] devised a novel method of molecularly imprinted polymer (MIP) and aptamers (Apt) for ultra-trace detection of the enzyme trypsin (Trp). The GCE was modified by electrochemical deposition of NiO nanoparticles with a complex containing aptamers and trypsin (Apt-Trp). This work exhibited a linear range from 1 to 90 pg/mL and an LOD of 0.75 pg/mL.

**Figure 3:**
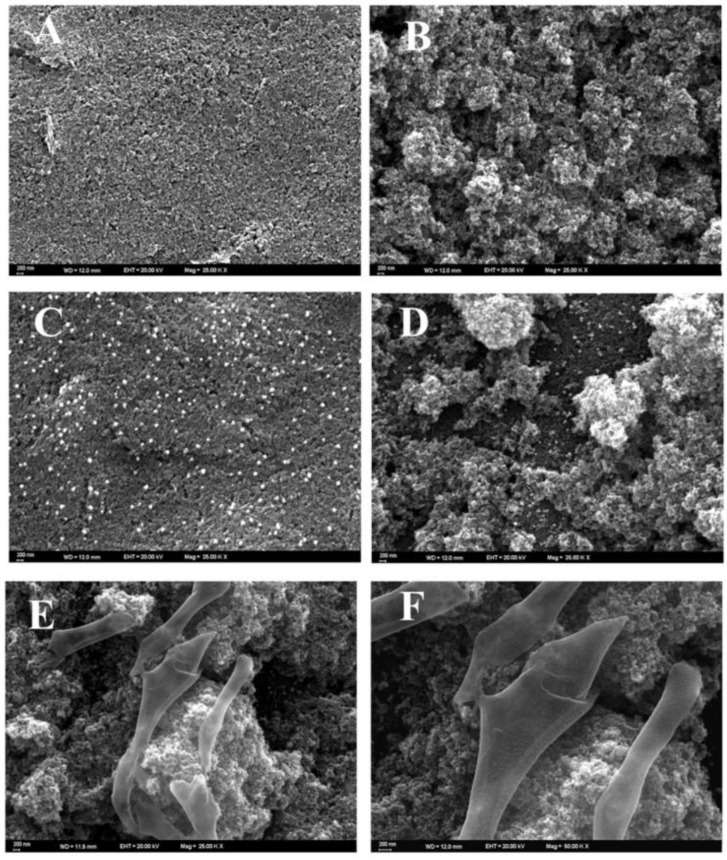
SEM images of (**A**) SPE, (**B**) TiO_2_ NPs/SPE, (**C**) Au NPs/SPE, (**D**) Au NPs/TiO_2_ NPs/SPE, (**E**) Apt/Au NPs/TiO_2_ NPs/SPE at 25.00 KX and (**F**) Apt/AuNPs/TiO_2_NPs/SPE at 50.00 KX [96]. Reprinted from Sensors and Actuators B, Vol/358, Gözde Aydoğdu Tiğ, Bengi Uslu. First label-free impedimetric aptasensor based on Au NPs/TiO2 NPs for the determination of leptin, 131420, 2022. Copyright (2023) with permission from Elsevier.

The impedimetric aptasensor for the determination of leptin was devised by Erkmen et al. [96]. The reported aptasensor was based on the SPE that has been enhanced with AuNPs and TiO_2_ nanoparticles (Figure 3) to detect leptin in human serum and plasma samples. A thiol DNA aptamer as a biorecognition element was bound onto the modified electrode surface. Two linear ranges were reported, the first from 1 to 100 pg/mL and the second from 100 to 1000 pg/mL. An LOD of 0.312 pg/mL was obtained.

Hossein Mashhadizadeh et al. [97] reported a carbon paste electrode (CPE) modified with magnetite and AuNPs to immobilize a thiol-modified hepatitis B virus (HBV) probe DNA and determine trace amounts of the target HBV DNA. The proposed DNA biosensor measured the viral target HBV DNA concentration with the LOD of 3.1 (±0.1) × 10^−13^ M, which was significantly lower than the LOD reported for gold or magnetite nanoparticles alone. The probe and target HBV DNA hybridization was measured by a change in the interfacial charge transfer resistance (*R*_ct_). The *R*_ct_ difference was linearly correlated with the complementary oligonucleotide concentrations from 8.3 (±0.1) × 10^−13^ to 6.4 (±0.2) × 10^−7^ M. The study was also effectively used for the impedimetric detection of target HBV DNA in urine and blood plasma samples.

Dinçkaya et al. developed [98] a DNA biosensor to detect aflatoxin M1 (AFM1). To immobilize a thiol-modified single-stranded DNA probe (ss-HSDNA) that binds to aflatoxin M1 explicitly, a self-assembling monolayer (SAM) of cysteamine and AuNPs was prepared layer-by-layer on Au electrodes. Cysteamine, AuNPs, and ss-HSDNA assembly processes were monitored by electrochemical impedance spectroscopy (EIS) and cyclic voltammetry (CV). The redox probe for the electrochemical studies was [Fe(CN)_6_]^−3/−4^ solution. With a standard deviation of 0.36 ng/mL, the biosensor exhibited a linear response to aflatoxin M1 across the concentration range of 1 to 14 ng/mL.

A probeless and label-free impedimetric biosensor for D-dimer (DD) was devised by Tasić et al. [99]. In order to attach a monoclonal antibody (mAb) against DD as a biorecognition element, AuNPs and dihexadecyl phosphate (DHP) were dispersed on the surface of screen-printed carbon electrodes (SPCEs). Measurements were again performed using EIS. To interpret the raw impedance spectra, two distinct analytical models were applied. The initial model was based on the complex capacitance value at a frequency of 200 mHz, which described the electrochemical occupancy of the DHP monolayer and its capacitive response at this frequency. The second model, fitted by two different Randles equivalent circuits, was based on the changes in *R*_ct_ that occur upon binding between mAb and DD. The models were compared, and a potential point-of-care (POC) sensor displayed a high linearity in the clinically relevant range of 500 ng/mL as well as an LOD of 8.92 ng/mL.

A polyorthophenylenediamine substrate with AuNPs and ss-DNA aptamers immobilized on the surface of a pencil graphite electrode (PGE) for detecting and determining plasma insulin was devised by Ensafi et al. [100]. EIS and CV were utilized for the characterization of the electrochemical biosensor. The CV was employed for the polyorthophenylene film coating formation via the electropolymerization process, and the EIS was for the evaluation of the charge transfer resistance of this aptasensor. This biosensor obtained performance metrics that yield a linear range of 1 to 1000 nmol/L with an LOD of 0.27 nmol/L.

An impedimetric immunosensor for the detection of viruses was devised by El Muttaqien et al. [101]. In this work, polyaniline (PAni) was electrostatically bound to AuNPs, which were then deposited on the surface of the gold electrode. The sensor was developed for the detection of model viruses called norovirus-like particles (NoV-LPs). The sensor showed a low LOD of 1.80 fg/mL. Moreover, the anti-dengue virus (DENV) non-structural protein 1 (NS1) was used to demonstrate the adaptability of the sensor and evaluate its application. This sensor successfully detected clinical DENV NS1 samples from patients and distinguished between positive and negative dengue infections. Furthermore, this work exhibited a very wide linear range in comparison to other studies.

Zhao et al. [102] devised an AChE-based biosensor that used metallic MoS_2_ nanosheets as the detection platform for paraoxons. This biosensor demonstrated outstanding electrocatalytic performance towards the electrochemical oxidation of thiocholine using ATCh as the substrate. The obtained results showed that the developed biosensor exhibited linearity with a paraoxon concentration range of 1.0 to 1000 g/L and a low LOD of 0.013 g/L.

The biosensor developed by Parra-Alfambra et al. [103] was based on MoS_2_ nanosheets obtained by an exfoliation process on the surface of a GC electrode, together with the enzyme lactate oxidase (LOx). EIS investigated the charge transfer process at the electrode interface. The GC/MoS_2_/LOx biosensor was used for the determination of lactate in the presence of hydroxymethylferrocene (HMF) as a redox mediator. From the calibration curve, a linear concentration range of 0.056 to 0.77 mM and an LOD of 0.17 μM were obtained.

**Table 1:** Metal and metal oxide and two-dimensional transition metal dichalcogenides-modified impedimetric biosensors.

Analyte	Recognition Element	Electrode	Linear Range	LOD	Reference
CRP	Nitrocellulose membrane	Nano-ZnO/CuO membranes	/	0.027 ng/mL	[94]
Trypsin	Molecularly imprinted polymer and aptamers	GCE/NiO/Apt-ePDA/MIP	10^−3^ to 9 × 10^−2^ ng/mL	7.5 × 10^−4^ ng/mL	[95]
Leptin	Thiol DNA aptamer	AuNPs/TiO_2_ NPs/SPE	10^−3^ to 10^−1^ ng/mL, 10^−1^ to 1 ng/mL	3.12 × 10^−4^ ng/mL	[96]
Target HBV DNA	Probe HBV DNA	CPE-magnetite-AuNPs	8.3 (±0.1) × 10^−4^ to 6.4 (±0.2) × 10^2^ nM	3.1 (±0.1) × 10^−4^ nM	[97]
AFB M1	ss-HSDNA	SAM of cysteamine and AuNPs-Au-electrode	1 to 14 ng/mL	/	[98]
D-dimer	DD antibody	AuNPs DHP SPCE	5 × 10^2^ ng/mL	8.92 ng/mL	[99]
Plasma insulin	ss-DNA aptamer	AuNPs PGE	10 to 10^3^ nM	2.7 × 10^8^ nM	[100]
NoV-LPs	Anti-NoV antibody	PAni/AuNPs/Au electrode	10^−4^ to 10^3^ ng/mL	1.8 × 10^−6^ ng/mL	[101]
Paraoxon	AChE	MoS_2_ nanosheets	10^6^ to 10^9^ ng/mL	1.3 × 10^4^ ng/mL	[102]
Lactate	Lactate oxidase	GC/MoS_2_	5.6 × 10^4^ to 7.7 × 10^5^ nM	1.7 × 10^4^ nM	[103]

### 3.2. Graphene and Graphene Oxide (GO)

A label-free impedimetric biosensor based on polyaniline (PANI) and graphene (G) composite at the GCE for the detection of *Escherichia coli* type DH5α with lectin concanavalin (ConA) as the biorecognition element was devised by Yang et al. [104] (Table 2). This study reported that the *R*_ct_ increased when the *E. coli* concentration increased linearly from 5.0 × 10^1^ cells/mL to 1.0 × 10^4^ cells/mL. The LOD of this impedimetric biosensor was reported to be 43 cells/mL. The developed biosensor shows a promising approach for the sensitive determination for the desired bacteria by incorporating a nanocomposite.

An electrochemical impedance biosensor based on graphene-modified GCE was developed by Asadi et al. [105] for the detection of miRNA-21, a known biomarker for early-stage prostate cancer. The biosensor showed a linearity ranging from 10^−14^ to 10^−8^ M with an LOD of 3 × 10^−15^ M. Moreover, in this study, the selectivity of the biosensor was investigated against non-complementary miRNA-141, and the devised biosensor exhibited good reproducibility, renewability, and stability, which suggested that the developed biosensor could present an alternative to conventional methods in early clinical diagnosis.

Wu et al. [106] developed an impedimetric biosensor from a self-assembled PTCA-rGO composite film. To produce a thrombin aptasensor, an in situ layer-by-layer self-assembly method was used to gradually deposit rGO, 3, 4, 9, 10-perylene tetracarboxylic acid (PTCA) and thrombin aptamer (TBA) onto a 3-aminopropyltriethoxy silane (APTES)-modified GCE. The prepared GCE/rGO/PTCA/TBA sensor exhibited a wide linear range from 1 pM to 100 nM, an LOD of 0.2 pM, and good specificity for thrombin.

For the impedimetric detection of the glycoprotein invertase (INV), Filip et al. [107] immobilized concanavalin A (ConA) lectin on an electrochemical rGO/thionine (Thi) surface by glutaraldehyde (GA) cross-linking. The immobilization of ConA/GA to the rGO/Thi surface resulted in a developed biosensor with a linear response in the concentration range from 10^−14^ to 10^−8^ mol of INV.

**Figure 4:**
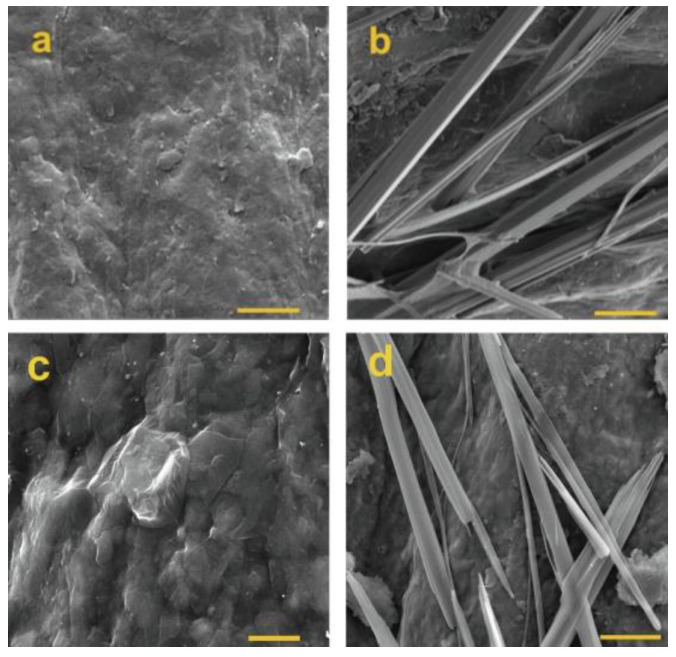
SEM images of (**a**) non-modified PGE, (**b**) PNT-GO, (**c**) GO, and (**d**) PNT-deposited PGE (Scale bar: 10 µm for (**a**,**b**,**d**) and 2 µm for (**c**)) [108]. Reprinted from Microchemical Journal, Vol/166, Gulcin Bolat, Oznur Akbal Vural, Yesim Tugce Yaman, Serdar Abaci. Label-free impedimetric miRNA-192 genosensor platform using graphene oxide decorated peptide nanotubes composite, 106218, 2021. Copyright (2023) with permission from Elsevier.

In order to quantify diabetic nephropathy and the cancer-related miRNA-192, Bolat et al. [108] devised a sensing platform using diphenylalanine-based self-assembled peptide nanotubes (PNTs) with GO on PGEs (Figure 4).

**Figure 5:**
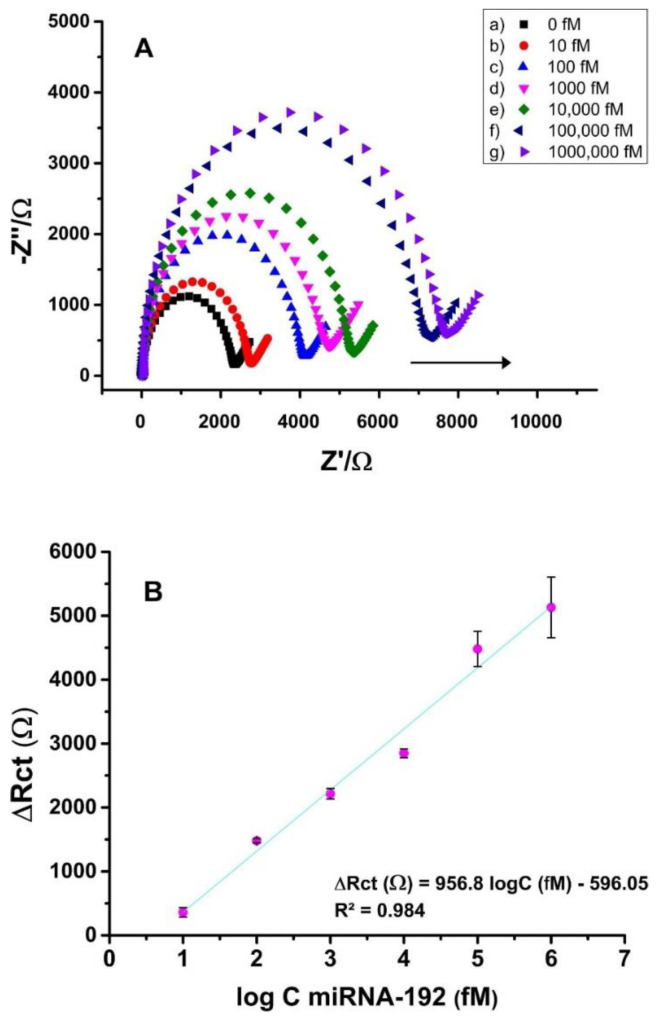
(**A**) The EIS response of PNT-GO-based genosensor at different target miRNA concentrations: 0 fM, 10 fM, 100 fM, 1 pM, 10 pM, 100 pM, and 1 nM of miRNA-192 and (**B**) plot of the corresponding measured Δ*R*_ct_ values versus the logarithm of target concentration [108]. Reprinted from Microchemical Journal, Vol/166, Gulcin Bolat, Oznur Akbal Vural, Yesim Tugce Yaman, Serdar Abaci. Label-free impedimetric miRNA-192 genosensor platform using graphene oxide decorated peptide nanotubes composite, 106218, 2021. Copyright (2023) with permission from Elsevier.

Based on the PNT-GO characteristics, the measured impedance was applied to assess the degree of hybridization between miRNA-192 and the associated ss-DNA probe (Figure 5). For miRNA-192 electrochemical analysis, PNT-GO/PGE demonstrated a good sensitivity with a broad detection range from 10 fM to 1.0 nM with an LOD of 8 fM.

### 3.3. Carbon Nanotubes (CNTs)

**Figure 6:**
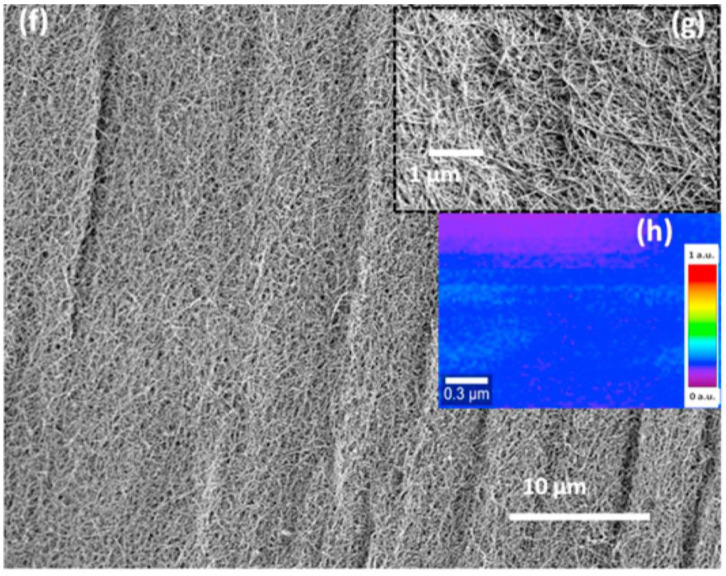
**A** SEM figure representing CNT aerogel modified electrode surface [109]. Reprinted from Biosensors and Bioelectronics, Vol/191, Jyoti Prakash, Anusree Dey, Sheetal Uppal, Rajath Alexander, Amit Kaushal, Hari Sharan Misra, Kinshuk Dasgupta. Label-free rapid electrochemical detection of DNA hybridization using ultrasensitive standalone CNT aerogel biosensor, 113480, 2021. Copyright (2023) with permission from Elsevier.

Carbon nanotubes (CNTs) have garnered significant interest in the development of impedimetric biosensors due to their unique electrical, mechanical, and structural properties. For example, Prakash et al. [109] reported a label-free DNA biosensor with an integrated standalone CNT aerogel electrode (Table 3) for SARS-CoV-2 nucleic acid detection. It was reported that the tenuous multi-directional network of clustered CNT embedding into the CNT aerogel electrode (Figure 6) provided high sensitivity and showed linear ohmic and almost isotropic electrical characteristics. Hybridization of the target DNA was identified by measuring a quantifiable change in electrochemical impedance that responds either specifically to the single-stranded target probe alone or to the double-stranded target probe complex. The LOD of this biosensor was determined at 1 pM.

Furthermore, Jiang and Lee [110] prepared a composite of MWCNT and polydimethylsiloxane and used it as an electrode for DNA sensing by EIS. An advantage of this electrode is reportedly that it also serves as a DNA recognition layer by forming π–π interactions between the MWCNTs and DNA without modifying the surface or immobilizing the probe, as is often the case with other electrodes. It was reported that the developed electrode is easily reusable by a simple cleaning procedure since there are no covalently bonded adsorbates on the electrode. This system could detect the mismatch of each base between the target and the probe with an LOD of 25 pM. Results show that EIS is promising for using the MWCNTs and polydimethylsiloxane electrodes as DNA sensors.

MWCNTs were also employed by Ensafi et al. [111] to identify DNA damage induced by mitomycin C (MCC). In this research, a pretreated pencil graphite electrode (PGE) modified with MWCNTs ranging between 70 and 110 nm and poly diallyl dimethylammonium chloride (PDDA), with adhered ds-DNA as a biorecognition element was employed. Electrodes were prepared by PDDA, a water-soluble, quaternary ammonium and cationic polyelectrolyte that acts as a positively charged colloid when dissolved in aqueous solutions. Herein, it was applied as a dispersant of MWCNTs. EIS measurements were performed in the presence of [Fe(CN)_6_]^−3/−4^ at a polarization potential of 0.10 V and the frequency range of 5 × 10^5^ to 10^−3^ Hz and at an amplitude of 10 mV. This study suggests that ds-DNA alkylation and cross-linking may have occurred during MMC reduction.

Singh et al. [112] reported that CNTs grown in situ at low temperatures on photolithographically defined Au microelectrode arrays printed on a glass substrate (CNTs/Au MEA) can be used as a highly sensitive electrochemical sensor for glucose detection. The ability to monitor 64 samples separately to detect hyperglycemia is one of the main advantages of the current system. By immobilizing the enzyme glucose oxidase (GOx) in the matrix of poly(paraphenylenediamine) (GOx/poly(p-PDA)/CNTs/Au MEA), the modified CNTs/Au MEA was able to detect glucose selectively. CV and EIS were applied to investigate the electrocatalytic and electrochemical responses of the designed sensor platform to glucose detection. The developed impedimetric biosensor showed a linear response in a concentration range from 0.2 to 27.5 µM with an LOD of 0.2 ± 0.0014 μM.

### 3.4. Carbon Nanofibers (CNFs)

Erdem et al. [113] devised an aptasensor (Table 4) based on CNF-SPE to perform impedimetric detection of thrombin (THR). In this study, *R_ct_* was measured by EIS before and after the immobilization of a DNA aptamer (APT) that is selective for THR on the surface of CNF-SPE. The average *R*_ct_ value of the modified electrode was 192.2 ± 26.4 Ω, which is 50 times larger than that of the unmodified CNF-SPE. This increase in *R*_ct_ value was due to the repulsive forces between DNA APT’s negatively charged phosphate groups and the anionic [Fe(CN)_6_]^3−/4−^. Additionally, the change in *R*_ct_ indicated that APT was successfully immobilized on the surface of the electrode. Moreover, a DNA aptamer that is different from the THR-specific APT and a random DNA oligonucleotide were used to assess the aptasensor’s selectivity. In the fetal bovine serum (FBS) medium, impedimetric sensing of the target protein was investigated at various THR concentrations. Furthermore, the aptasensor’s selectivity for protein C (PC) and bovine serum albumin (BSA) was examined in the FBS medium. The reported CNF-SPE-based aptasensor facilitates reliable and selective impedimetric monitoring of THR. Compared to other CNF-based biosensors, this biosensor does not exhibit good sensitivity.

A phage-based electrochemical biosensor for the detection of *Escherichia coli* was devised by Wang and Wang [114]. In a two-step drop-casting procedure, electrospun CNFs derived from polyacrylonitrile were deposited on bare SPEs, followed by the immobilization of *E. coli* bacteriophages. Bacteriophage deposition was carried out on CNFs produced by carbonizing electrospun PAN nanofibers. The results indicated that the devised biosensor had an LOD of 36 CFU/mL in PBS, a linear range of 10^2^ to 10^6^ CFU/mL, and a response time of 10 min. The devised biosensor’s outstanding durability throughout 1 month of room temperature storage and an excellent sensitivity to the host bacteria compared to other pathogens in food were reported.

A poly (propylene imine) dendrimer-carbon nanofiber nanocomposite (CNFs-PPI) immobilization platform-based aptasensor for bisphenol A (BPA) was devised by Tsekeli et al. [115]. Glutaraldehyde was utilized as a crosslinker to create covalent bonds between amino-modified aptamers and the CNFs-PPI platform on a glassy carbon electrode (GCE). Using electrochemical impedance spectroscopy (EIS), differential pulse voltammetry (DPV), and cyclic voltammetry (CV), it was determined how the interfacial modifications at the electrode surface changed during the production and application of the biosensor. This aptasensor detected BPA linearly in the range of 1 to 10 nM with an LOD of 0.06 nM.

### 3.5. Quantum Dots (QDs)

Zhao et al. [89] devised an electrochemical biosensor (Table 5) for glucose detection, where the synergistic labeling strategy using colloidal PbS QDs and Au nanospheres (AuNSs) was applied. The one-step dip coating technique was used to show that the PbS CQDs/AuNSs/glucose oxidase (GOx) mixture could be permanently immobilized on the carbon electrode surface. The functionalities of particular molecular recognition, signal transduction, and signal amplification were incorporated into the electrochemical biosensor employing the PbS CQDs/AuNSs/GOx-modified electrode. The sensor was able to convert the glucose enzyme-catalyzed reaction into detectable current signals with a linear response in the glucose concentration range of 0.1 M to 10 mM and an LOD of 1.432 nM.

**Figure 7:**
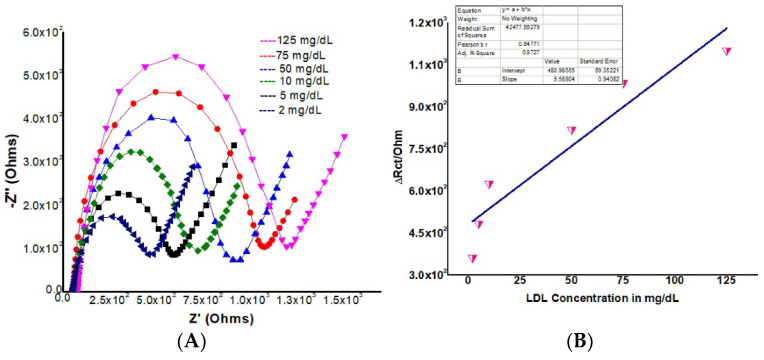
(**A**) Nyquist plot of AAB/rGO-MPA cCdSe QD/ITO immunoelectrode with different concentrations of LDL; (**B**) Δ*R*_ct_ vs. concentration of LDL in mg/dL [116]. Reprinted from Materials Letters, Vol/308, Part B, Drishti Khandelwal, Disha Jain, Pratima R Solanki, Kumar Rakesh Ranjan, Maumita Das Mukherjee. A novel nanocomposite platform of mercaptopropionic acid stabilized CdSe quantum dots-graphene for impedimetric detection of low-density lipoprotein, 131236, 2022. Copyright (2023) with permission from Elsevier.

Electrophoretically deposited, 3-mercaptopropionic acid-capped CdSe QDs-graphene nanocomposite (rGO-CdSe QDs/ITO) (Figure 7) were used to devise an electrochemical biosensor for the detection of low-density lipoprotein (LDL) by the immobilization of anti-apolipoprotein B (AAB) via N-ethyl-N′-(3-dimethylaminopropyl carbodiimide N-hydroxysuccinimide) coupling by Khandelwal et al. [116]. AAB/rGO-CdSe-QDs/ITO revealed a linear detection of LDL in the wide range of 2 mg/dL to 125 mg/dL with an LOD of 3.76 mg/dL by EIS.

Moazampour et al. [117] designed a label-free electrochemical genosensor based on ZnS QDs functionalized with L-cysteine (Cys-ZnS-QDs) to detect miR-200a, a specific biomarker for ovarian cancer. Cys-ZnS-QDs, which serve as a suitable substrate for immobilizing the DNA probe, were electrodeposited onto the GCE surface. The EIS was applied to evaluate the analytical capabilities of the genosensor. Optimal effective parameters were used in the design of the genosensor to maximize the analytical performance. The linear range and the LOD of miR-200a were found to be from 1.0 × 10^−14^ to 1.0 × 10^−6^ M and 8.4 fM, respectively. Moreover, the miRNA strand with the base mismatch was detected by the genosensor separately from the corresponding miRNA target strand.

SARS-CoV-2 protein biosensor using colloidal quantum dots (CQDs)-modified electrode was devised by Zhao et al. [118]. The distinctive current peak correlated with the precise binding reaction of the antibody and SARS-CoV-2 antigen proteins. Quantum confinement, Coulomb blockade, and quantum tunneling effects of quantum dots were thought to be the cause of the unusual charging and discharging action, which was dependent on the applied AC voltage. The specific binding reaction between the antigen and the antibody could be detected by the electrode modified with CQDs, and it could subsequently be converted into a significant electrical current. With a correlation coefficient of 93.8% when compared to ELISA data, the all-solid-state protein biosensor enables the quantitative measurement of SARS-CoV-2 antibodies in serum samples from COVID-19 patient samples. The linearity and LOD of the concentration-response curve were calculated to be from 0.969 to 4.99 ng/mL, and 7.73 mg/mL.

## 4. Conclusions

In the last decade, interest in nanosized particles has drastically increased due to their physical and chemical properties [119]. Nanoparticle biosensors can potentially improve or replace current analytical techniques, and their introduction could have significant implications for both research and clinical practice [46]. In electroanalytical chemistry, many materials have been studied with respect to their possible role in the nanoparticle modification of an electrode. The ease of this modification, stability, and broad analytical application of these materials led to significant research on subtly different nanoparticle deposits devised by many particular production techniques.

As reviewed in this work, nanoparticles are utilized in various fields, including detecting viruses and microorganisms, medical parameters, environmental pollutants, and toxins. The most commonly used nanoparticles represent metal nanoparticles, where AuNPs are often employed in developing electrochemical biosensors due to their stability and biocompatibility. Moreover, carbon nanotubes, carbon nanofibers, graphene oxide, quantum dots, and metal nanoparticles all exhibit unique properties that make them potentially applicable in impedimetric biosensors. The choice of material will depend on the specific application and the desired properties of the biosensor.

Many challenges still remain in developing and applying nanomaterial-based impedimetric biosensors. Choosing the right nanomaterial for the intended application and synthesizing them consistently with desired properties is complex and requires expertise in material science. Attaching biorecognition elements to nanomaterials while maintaining their activity and stability can be challenging. Furthermore, avoiding and recognizing matrix effects in complex samples is important, where components other than the target analyte influence biosensor response. Addressing these challenges requires interdisciplinary collaboration, advanced nanomaterial synthesis techniques, characterization methods, and innovative impedimetric biosensor design. Future research will focus on improving the sensitivity and selectivity of nanomaterial-based impedimetric biosensors and on the development of novel nanomaterials, such as biodegradable nanomaterials.

Overall, using nanoparticles in impedimetric biosensors can improve their selectivity, sensitivity, and response times, making them more practical for a wide range of applications, such as medical diagnostics, environmental monitoring, and food safety testing.

## Figures and Tables

**Table 2:** Graphene- and graphene oxide-modified impedimetric biosensors.

Analyte	Recognition Element	Electrode	Linear Range	LOD	Reference
*E. coli* DH5α	ConA	PANI/G/GCE	5.0 × 10 to 1.0 × 10^4^ cells/mL	43 cells/mL	[104]
ss-DNA	miRNA-21	graphene-modified GCE	10^−5^ to 10 nM	3 × 10^−6^ nM	[105]
Thrombin	Aptamer	GCE/rGO/PTCA/TBA	10^−3^ nM to 10^2^ nM	2 × 10^−4^ nM	[106]
INV	ConA	ErGO/Thi	10^−6^ to 10 nM	/	[107]
miRNA-192	ss-DNA probe	PNT-GO/PGE	10^−5^ to 1 nM	8 × 10^−5^ nM	[108]

**Table 3:** CNT-modified impedimetric biosensors.

Analyte	Recognition Element	Electrode	Linear Range	LOD	Reference
SARS-CoV-2 ss-DNA	Complimentary ss-DNA	CNT aerogel electrode	10^−1^ to 10^3^ nM	10^−3^ nM	[109]
O-ss-DNA A-ss-DNA	Complimentary ss-DNA	MWCNT-polydimethylsiloxane electrodes	5 × 10^−2^ to 10 nM	2.5 × 10^−2^ nM	[110]
MMC	ds-DNA	MWCNT-PGE	/	/	[111]
Glucose	GOx	CNTs/Au MEA	2 × 10^2^ to 2.75 × 10^4^ nM	2 × 10^2^ ± 1.4 nM	[112]

**Table 4:** CNF-modified impedimetric biosensors.

Analyte	Recognition Element	Electrode	Linear Range	LOD	Reference
THR	DNA APT	CNF-SPE	5 × 10^3^ to 2 × 10^4^ ng/mL	1.8 × 10^4^ ng/mL	[113]
*E. coli*	Bacteriophage	CNF-SPE	10^2^ to 10^6^ CFU/mL	36 CFU/mL	[114]
BPA	NH-aptamer	GCE/CNFs-PPI	1 to 10 nM	6 × 10^−2^ nM	[115]

**Table 5:** Quantum dots-modified impedimetric biosensors.

Analyte	Recognition Element	Electrode	Linear Range	LOD	Reference
Glucose	Glucose oxidase	PbS CQDs/AuNPs/GOx	10^2^ to 10^7^ nM	1.432 nM	[89]
LDL	AAB	rGO-CdSe QDs/ITO	2 × 10^7^ to 1.25 × 10^5^ ng/mL	3.76 × 10^7^ ng/mL	[116]
miR-200a	L-cysteine	Cys-ZnS-QDs	10^−5^ to 10^3^ nM	8.4 × 10^−6^ nM	[117]
SARS-CoV-2 antibodies	SARS-CoV-2 protein	CQDs	0.969 to 4.99 ng/mL	7.73 ng/mL	[118]

## Data Availability

Not applicable.

## Abbreviation

**Abbreviation**	**Definition**
AAB	Anti-apo lipoprotein B
AC	Alternating current
APTMS	3-aminopropyltrinemethoxysilane
Au-IDE	Gold interdigitated microelectrode
AuNPs	Gold nanoparticles
Au-SPE	Gold surface-screen printed electrode
BN	Boron nitride
BPA	Bisphenol A
CBZ	Carbendazim
C_dl_	Double-layer capacitance
CE	Counter electrode
CNF	Carbon nanofibers
CNT	Carbon nanotube
ConA	Concanavalin A
CPE	Carbon paste electrode
CPR	C-reactive protein
CQD	Colloidal quantum dots
CV	Cyclic voltammetry
Cys	Cysteine
DD	D-Dimer
DHP	Dihexadecyl phosphate
EC	Capillary electrophoresis
EEC	Equivalent electric circuit
EIS	Electrochemical impedance spectroscopy
ELISA	Enzyme-linked immunosorbent assay
ErGO	Electrochemical-reduced graphene oxide
GCE	Glassy carbon electrode
GO	Graphene oxide
GOx	Glucose oxidase
GQD	Graphene quantum dots
HBV	Hepatitis B virus
HPLC-MS	High-performance liquid chromatography-mass spectroscopy
HPR	Horseradish peroxidase
ITO	Indium tin oxide
LDL	Low-density lipoprotein
LOD	Limit of detection
MWCNT	Multi-walled carbon nanotube
PDDA	Poly diallyl dimethylammonium chloride
PGE	Pencil graphite electrode
POC	Point-of-care
QD	Quantum dots
*R*_ct_	Charge transfer resistance
RE	Reference electrode
rGO	Reduced graphene oxide
R_s_	Ohmic resistance
SAM	Self-assembled monolayer
SARS-CoV	Severe acute respiratory syndrome coronavirus
SPCE	Screen-printed carbon electrode
ss-HDNA	Thiol-modified single-stranded DNA
SWCNT	Single-walled carbon nanotube
SWV	Square wave voltammetry
THR	Thrombin
TLC	Thin-layer chromatography
WE	Working electrode
Z_w_	Warburg impedance
